# Diagnosis of plantar vein thrombosis by vascular ultrasound: a case report

**DOI:** 10.1590/1677-5449.202400812

**Published:** 2025-06-16

**Authors:** Mariana Jordão França, Luciana Akemi Takahashi, Graciliano José França

**Affiliations:** 1 Universidade Positivo (UP), Curitiba, PR, Brazil.; 2 Universidade Federal do Paraná (UFPR), Curitiba, PR, Brazil.

**Keywords:** Doppler ultrasonography, lower limb, venous thrombosis, edema, vascular system injuries, case reports

## Abstract

Plantar vein thrombosis is a vascular disease that affects the medial and/or lateral plantar veins. Its clinical manifestations are generally non-specific. However, it usually presents with pain in the plantar region, edema, and difficulty walking. The disease predominantly affects middle-aged females and is idiopathic in the majority of cases. The most commonly associated risk factors are recent surgical procedures, cancer, use of oral contraceptives, local trauma, and genetic disorders, such as hereditary thrombophilia. The gold standard diagnostic test is vascular ultrasound, evaluating venous compressibility. We report a case of venous thrombosis of the lateral plantar vein diagnosed with vascular ultrasound.

## INTRODUCTION

The venous plexus of the foot starts in the veins of the toes, which give origin to the plantar digital veins. These join to form the metatarsal veins, which are located between the metatarsal spaces and form the deep plantar arch, located in the proximal forefoot. The deep plantar arch drains to the lateral and medial plantar veins. The lateral plantar vein accompanies the lateral plantar artery and is located between the flexor digitorum brevis and the quadratus plantae muscles. The medial plantar vein accompanies the medial plantar artery, coursing between the adductor hallucis and the flexor hallucis brevis muscles. The lateral and medial plantar veins give off the veins of the superficial great and small saphenous systems and join behind the medial malleolus to form the deep posterior tibial veins.

The plantar venous system and the calf muscle pumps are essential components in venous return of the lower limbs. It is estimated that the plantar veins eject around 25 ml of blood with every step walked.^1^

Plantar vein thrombosis (PVT) is a rare condition. Its clinical manifestations are nonspecific, but it generally presents with pain in the plantar region that can worsen with walking and may be associated with edema.^2^

Since plantar pain is a common complaint, accounting for around 11 to 15% of complaints related to the feet and ankles, several possible diagnoses should be investigated, such as, for example, plantar fasciitis, which is the most common cause.^3^ Regardless, other etiologies, such as tendinopathies, ganglion cysts, crystal deposition disease, Morton’s neuroma, intermetatarsal bursitis, sesamoiditis, and stress fracture should also be ruled out.^1^

Vascular ultrasound (VUS) is considered the gold-standard examination for diagnosis of PVT. Characteristic findings are an absence of vein compressibility, a hypoechoic image suggestive of intraluminal thrombus, and absent blood flow on Doppler.^2^ Magnetic resonance imaging (MRI) can also be ordered if other differential diagnoses are suspected.^4^

The etiology of PVT is unknown and fewer than 100 cases are described in the literature.^5^

This study was approved by the Ethics Committee at our institution (decision number 6.860.302). A free and informed consent form for studies involving human beings was signed. The patient signed a free and informed consent form granting permission for publication of ultrasound images and the case description.

## CASE DESCRIPTION

The patient was a 65-year-old female with no comorbidities who presented with pain in the right plantar region and local edema with onset 4 days previously. She had consulted an orthopedic specialist 2 days before and had been diagnosed with plantar fasciitis. However, the pain had not improved with ibuprofen and she had returned to the physician, who referred her to the Vascular Surgery Service for VUS of the right lower limb to rule out or confirm deep venous thrombosis.

**Figure 1:**
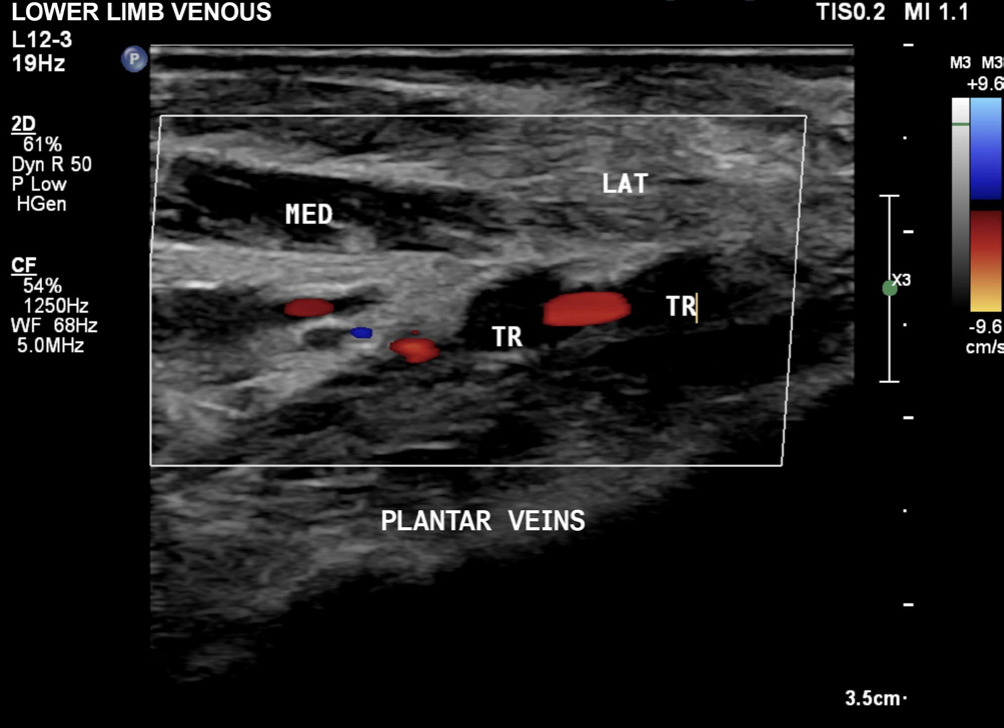
Plantar vascular ultrasound of the right lower limb, in the transverse plane, showing venous thrombosis of the lateral plantar vein. TR = thrombus; MED = medial compartment; LAT = lateral compartment.

**Figure 2:**
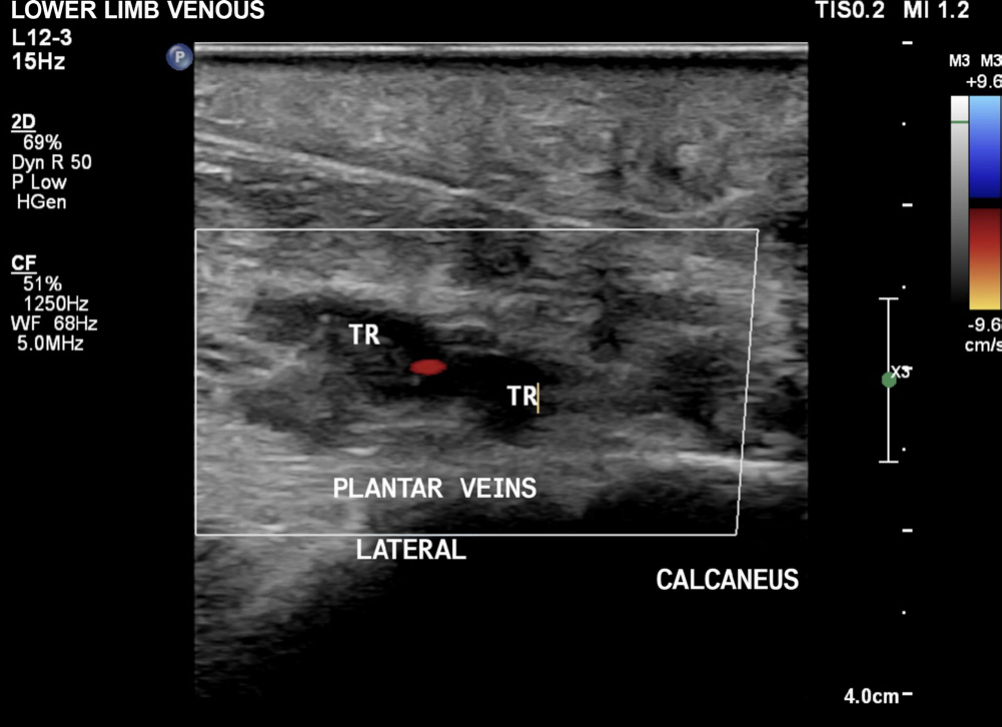
Plantar vascular ultrasound of the right lower limb, in the transverse plane, showing venous thrombosis of the lateral plantar vein. TR = thrombus.

The VUS examination showed normal femoral, popliteal, anterior tibial, posterior tibial, fibular and gastrocnemius veins. However, abnormalities of the lateral plantar veins were observed that were compatible with recent occlusive thrombosis (Figures 1 and 2), characterized by incompressibility of the segment, absence of flow on Doppler, and presence of images suggestive of intraluminal thrombus.

**Figure 3:**
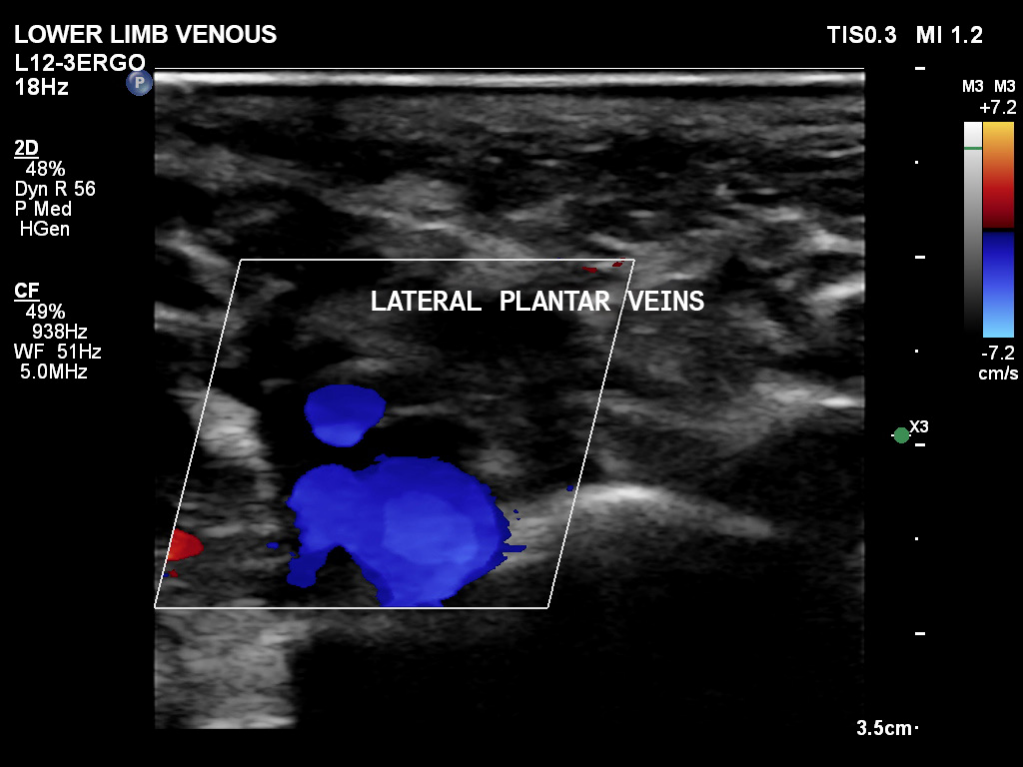
Control plantar vascular ultrasound of the right lower limb at 3 months, in transverse view, demonstrating complete recanalization of the prior thrombus.

In view of this, treatment was started with 500 mg acetaminophen four times a day, until resolution of the pain, and 15 mg rivaroxaban twice a day for 3 weeks, followed by a maintenance dose of 20 mg a day, for 3 months. The patient exhibited clinical improvements, with reduced pain and edema after 3 days. The patient attended a consultation 3 months after starting anticoagulation and was free from complaints. A control examination was ordered, which showed the lateral plantar veins were patent, compressible, with no irregularities of the walls and no signs of reflux, suggestive of complete recanalization of the venous thrombosis (Figure 3).

## DISCUSSION

In 96% of cases, PVT occurs in the lateral plantar vein, because of its more superficial location in the plantar region, making it more susceptible to mechanical trauma.^1^ Plantar vein thrombosis is more common in females and mean age of incidence is 58.2 years.

Risk factors associated with PVT include hereditary and/or acquired thrombophilias, cancer, post-surgical immobility, mechanical stress, coronavirus infection, and/or oral contraceptives,^1,4^ especially third-generation forms containing desogestrel, gestodene, or norelgestromin.^6^ However, the majority of cases are described as idiopathic.^7^

The signs and symptoms of PVT include pain of moderate to high intensity involving the plantar surface of the foot, worsening when walking, and with or without edema. Presence of clubbing in the plantar region is a characteristic observed during the physical examination that increases the likelihood of this diagnosis.

Complications of PVT include extension of the thrombus to the deep vein system of the leg and pulmonary thromboembolism. Recurrence is not uncommon, occurring in up to 27% of cases.^5^

There is no consensus in the literature on the best treatment for PVT.^2^ The American College of Thoracic Surgeons recommends anticoagulation with heparin and vitamin K antagonists, or direct oral anticoagulants (rivaroxaban, apixaban, edoxaban, or dabigatran) for 3 months to 6 months, or indefinitely if the patient has thrombophilia, for example.^8^ Other possible treatments include non-steroidal anti-inflammatory drugs and analgesics and graduated compression elastic stockings.^4^ According to the Brazilian Society of Angiology and Vascular Surgery, the objective of continuing anticoagulation is to avoid PVT recurrence, the risk of which is lower in cases with reversible factors such as surgery, or higher in cases of idiopathic PVT or patients with cancer. For initial treatment, anticoagulation is recommended for 3 to 6 months, with direct oral anticoagulants the first choice for patients with no need for hospital admission.^9^

Laboratory tests such as D dimer can help to rule out PVT, since it has high sensitivity but low specificity.^10^

The examination of choice to confirm diagnosis is VUS, which suggests thrombosis in the presence of dilation and incompressibility of the vein, reduced or absent flow on Doppler, and presence of intraluminal thrombus.^11^

The most common differential diagnosis is plantar fasciitis, which is the most common cause of plantar pain and can be identified on ultrasound by the hypoechoic plantar fascia with thickness exceeding 4.5 mm and loss of the normal fibrous echotexture. In the case of Morton’s neuroma, there will be presence of a well-defined oval mass with variable echogenicity in contact with the long access of the interdigital nerve.^1^

An MRI can also be ordered to investigate other differential diagnoses or for obese patients, in whom VUS has technical limitations. A diagnosis of PVT is confirmed on T1 images in the presence of filling defects after administration of gadolinium contrast and hyper intense veins on T2 images. Discrete perivascular edema may also be observed in the muscles adjacent to the plantar veins. In the absence of thrombosis, the plantar veins will be hyperintense on T2. In stress fracture cases, the examination will show periosteal edema involving the marrow and adjacent soft tissues. An MRI scan can also diagnose ganglion cysts, in which case a uniformly hyperintense mass will be observed in T2 and walls highlighted after administration of gadolinium contrast. In sesamoiditis, the image will show marrow edema in the acute phase and sclerosis and reduced volume in the chronic phase. On MRI, crystal deposition diseases will show erosions, chondral defect, and periarticular edema with hypointense areas on T1 and T2 images suggestive of hydroxyapatite deposits. Intermetatarsal bursitis manifests hypointense T1 images and hyperintense images on fat-suppression T2, and, after administration of gadolinium, peripheral highlighting may be observed. Tendon injuries are revealed by enlarged tendon and abnormalities such as high intensity around the tendon and peritendon soft tissues.^1^

Patients with any type of history of prior thrombotic episode or who complain of pain in the plantar region should be carefully reassessed because of the high risk of recurrence.
