# Supplementary material for: Diagnosis of plantar vein thrombosis by vascular ultrasound: a case report
Source: J Vasc Bras. 2025 Jun 16;24:e20240081. doi: 10.1590/1677-5449.202400812 (PMC12186684; doi:10.1590/1677-5449.202400812)
Supplement: Translation

# Translation: Trombose de veia plantar diagnosticada por ultrassom vascular: um relato de caso

**DOI:** 10.1590/1677-5449.202400811

**Authors:** Mariana Jordão França, Luciana Akemi Takahashi, Graciliano José França

**Affiliations:** 1 Universidade Positivo – UP, Curitiba, PR, Brasil.; 2 Universidade Federal do Paraná – UFPR, Curitiba, PR, Brasil.

**Keywords:** ultrassonografia Doppler, membro inferior, trombose venosa, edema, lesões do sistema vascular, relatos de casos

## Abstract

A trombose de veias plantares é uma doença vascular que acomete as veias plantares laterais e/ou mediais. Suas manifestações clínicas geralmente são inespecíficas, porém, habitualmente cursam com dor na região plantar, edema e dificuldade na deambulação. A doença afeta predominantemente indivíduos do sexo feminino, de meia-idade e, na maioria dos casos, é idiopática. Os fatores de risco mais comumente associados são procedimentos cirúrgicos recentes, neoplasias, uso de anticoncepcionais orais, trauma local e desordens genéticas, como, por exemplo, trombofilias hereditárias. O exame diagnóstico padrão-ouro é a ultrassonografia vascular, que avalia a compressibilidade venosa. Reportamos um caso de trombose venosa de veia plantar lateral diagnosticada pelo ultrassom vascular.

## INTRODUÇÃO

O plexo venoso dos pés tem início nas veias dos plexos dos dedos, as quais dão origem às veias digitais plantares. Estas unem-se para formar as veias metatarsais, que se localizam entre os espaços metatarsais e formam o arco venoso profundo, situado no antepé proximal. O arco venoso profundo drena para as veias plantares lateral e medial. A veia plantar lateral acompanha a artéria plantar lateral e está localizada entre o músculo flexor curto dos dedos e o quadrado plantar. Já a veia plantar medial acompanha a artéria plantar medial, percorrendo o trajeto entre os músculos abdutor do hálux e flexor curto do hálux. As veias plantares lateral e medial emitem as veias do sistema superficial safenas parva e magna, e unem-se atrás do maléolo medial para formar as veias profundas tibiais posteriores.

O sistema venoso plantar e as bombas impulsoaspirativas são componentes fundamentais no retorno venoso dos membros inferiores. Estima-se que, a cada passo em uma caminhada, as veias plantares ejetem cerca de 25 ml de sangue^1^ .

A trombose das veias plantares (TVP) é uma condição rara. Suas manifestações clínicas são inespecíficas, porém, geralmente cursam com dor na região plantar, que pode piorar com a deambulação e estar associada a edema^2^ .

Como a dor na região plantar é uma queixa comum, representando cerca de 11 a 15% das queixas relacionadas aos pés e tornozelos, devem ser aventados alguns diagnósticos, como, por exemplo, a fascite plantar, a sua causa mais comum^3^ . Porém, outras etiologias, como tendinopatias, cistos gangliônicos, doença por depósito de cristais, neuroma de Morton, bursite intermetatarsal, sesamoidite e fratura por estresse devem ser afastadas^1^ .

O ultrassom vascular (UV) é considerado o exame padrão-ouro no diagnóstico de TVP. Os achados característicos neste exame complementar são ausência da compressibilidade da veia, imagem sugestiva de trombo intraluminal hipoecoico e ausência de fluxo ao Doppler^2^ . A ressonância nuclear magnética (RNM) também pode ser solicitada em caso de suspeita de diagnósticos diferenciais^4^ .

A etiologia TVP é desconhecida, e existem menos de 100 casos descritos na literatura^5^ .

O estudo foi aprovado pelo Comitê de Ética de nossa instituição (parecer número 6.860.302). O termo de consentimento livre e esclarecido foi assinado para os estudos que envolvem seres humanos. O paciente assinou o termo de consentimento livre e esclarecido para a divulgação de imagens ultrassonográficas e publicação do caso.

## DESCRIÇÃO DO CASO

Paciente do sexo feminino, 65 anos de idade, sem comorbidades, apresentou queixa de dor na região plantar direita há quatro dias, associada a edema local. Referiu ter se consultado com ortopedista dois dias antes, sendo diagnosticada com fascite plantar. No entanto, relatou que não houve melhora da dor com o uso de ibuprofeno e retornou ao médico, que solicitou parecer ao Serviço de Cirurgia Vascular para realização de UV do membro inferior direito, a fim de excluir ou confirmar a suspeita de trombose venosa profunda.

O exame de UV demonstrou veias femoral, poplítea, tibial anterior, tibial posterior, fibulares e gastrocnêmias sem alterações. Entretanto, nas veias plantares laterais (Figuras 1 e 2), foram identificadas alterações compatíveis com trombose recente e oclusiva, caracterizadas pela incompressibilidade desse segmento, ausência de fluxo ao Doppler e presença de imagem sugestiva de trombo intraluminal.

**Figura 1:** Ultrassom vascular plantar do membro inferior direito, em corte transversal, demonstrando trombose venosa da veia plantar lateral. TR = trombo; MED = compartimento medial; LAT= compartimento lateral.

**Figura 2:** Ultrassom vascular plantar do membro inferior direito, em corte transversal, demonstrando trombose venosa na veia plantar lateral. TR = trombo.

Diante disso, foi iniciado tratamento com acetaminofeno 500 mg, quatro vezes ao dia, até regressão da dor, e rivaroxabana 15 mg, duas vezes ao dia por três semanas, seguida de dose de manutenção de 20 mg ao dia, durante três meses. A paciente apresentou melhora clínica da dor e redução do edema após três dias. Três meses após o início da anticoagulação, foi realizada uma consulta, na qual a paciente se apresentou sem queixas. Foi solicitado um exame de controle, que evidenciou veias plantares laterais pérvias, compressíveis, sem irregularidades parietais e sem sinais de refluxo, sugestivo de recanalização completa da trombose venosa prévia (Figura 3).

**Figura 3:** Ultrassom vascular plantar do membro inferior direito de controle após três meses, em corte transversal, demonstrando recanalização completa do trombo prévio.

## DISCUSSÃO

Em 96% dos casos, a TVP ocorre na veia plantar lateral, devido à sua localização mais superficial na região plantar, o que a torna mais suscetível ao trauma mecânico^1^ . Além disso, é mais frequente no sexo feminino, e apresenta uma média de idade de 58,2 anos.

Os fatores de risco associados à TVP são trombofilias hereditárias e/ou adquiridas, neoplasias, imobilização pós-cirúrgica, estresse mecânico, infecção pelo coronavírus e/ou uso de contraceptivos orais^1,4^ , especialmente os de terceira geração contendo desogestrel, gestodeno ou norelgestromina^6^ . Porém, a maioria dos casos é descrita como idiopática^7^ .

Os sinais e sintomas da TVP incluem dor de moderada a alta intensidade na superfície plantar do pé, que pode piorar com a deambulação, associada ou não ao edema. A presença de empastamento da região plantar é uma característica observada no exame físico que aumenta a possibilidade do diagnóstico.

As complicações da TVP incluem a extensão do trombo para as veias do sistema venoso profundo da perna e o tromboembolismo pulmonar. A sua recorrência não é incomum, ocorrendo em até 27% dos casos^5^ .

Na literatura, não há um consenso para tratamento da TVP^2^ . O American College of Thoracic Surgeons recomenda o uso de anticoagulação com heparina e antagonista de vitamina K, ou o uso de anticoagulantes orais diretos (rivaroxabana, apixabana, edoxabana ou dabigatrana) por um período de três a seis meses, ou por tempo indeterminado se o paciente for portador de trombofilias, por exemplo^8^ . Outras possíveis terapias incluem anti-inflamatórios não esteroidais e analgésicos e o uso de meias elásticas de compressão graduada^4^ . De acordo com as diretrizes da Sociedade Brasileira de Angiologia e Cirurgia Vascular, o objetivo de prolongar a anticoagulação é evitar a recorrência de TVP, sendo esse risco menor em casos de fatores reversíveis, como cirurgia, e maior em TVP idiopática ou associada ao câncer. Para tratamento inicial, se sugere anticoagulação por três a seis meses, sendo os anticoagulantes orais diretos a escolha inicial em pacientes sem necessidade de internação^9^ .

Exames laboratoriais como dímero D podem auxiliar na exclusão do diagnóstico de TVP, por ser de alta sensibilidade e baixa especificidade^10^ .

O exame de escolha para confirmação diagnóstica é o UV, que sugere a presença de trombose pela dilatação e incompressibilidade da veia, redução ou ausência do fluxo no Doppler e presença de trombo intraluminal^11^ .

Em relação aos diagnósticos diferenciais, a fascite plantar, causa mais comum de dor na região plantar, pode ser caracterizada ao exame ultrassonográfico pela hipoecogenicidade e espessamento da fáscia plantar maior que 4,5 mm, associada à perda da ecotextura fibrilar normal. No caso do neuroma de Morton, haverá a presença de uma massa ovoide bem definida, com ecogenicidade variável e continuidade com o nervo interdigital no longo eixo^1^ .

A RNM também pode ser solicitada para avaliação de outros diagnósticos diferenciais ou em pacientes obesos, onde a UV apresenta limitações técnicas. O diagnóstico da TVP é confirmado na sequência T1 pela presença de defeitos de enchimento na imagem após administração do contraste de gadolínio, além da ausência de veias hiperintensas T2. Também pode ser observado discreto edema perivascular nos músculos adjacentes às veias plantares. Na ausência de trombose, as veias plantares são hiperintensas em T2. Em casos de fratura de estresse, o exame demonstra edema periosteal da medula e dos tecidos moles adjacentes. A RNM pode, também, diagnosticar cistos ganglionares, caso em que será observada a presença de uma massa uniformemente hiperintensa em T2 e paredes com realce após administração do contraste de gadolínio. Na sesamoidite, a imagem obtida revela edema medular na fase aguda e, na fase crônica, esclerose e redução de volume. Doenças por depósito de cristais apresentam, na RNM, erosões, defeito condral e edema periarticular associado a áreas hipointensas em T1 e T2, sugestivas de deposição de hidroxiapatita. A bursite intermetatarsal se manifesta com imagens hipointensas em T1 e hiperintensas em T2 com supressão de gordura e, após administrado o gadolínio, pode ser observado realce periférico. Já a tendinopatia é evidenciada no exame por aumento do tendão e presença de alterações, como alta intensidade ao redor do tendão e tecidos moles peritendíneos^1^ .

Pacientes com qualquer episódio trombótico prévio, com queixas de dor na região plantar, devem ser reavaliados com maior cautela devido ao alto risco de recorrência.
